# Scarlatina after Parturition

**Published:** 1859-10

**Authors:** 


*Scarlatina after Parturition.*—Dr. McClintock read a paper before the association of the fellows and licentiates of the King's and Queen's College of Physicians in Ireland (Feb. 2, 1859,) on the occurrence of scarlatina within eight days after confinement. The mortality in such cases has been put down as two out of three, or over sixty-six per cent. Ot twenty-eight patients treated in the Rotunda hospital, seven died, or about twenty-five per cent. Dr. McClintock considered the advent of this exanthem supervening on delivery one of the most fatal complications of the puerperal state. The earlier the appear ance of the rash, the more fatal; the same rule having applied to puerperal fever, whilst epidemic in this city in 1854 and 1855. He refered to the peculiar acceleration of the pulse in these cases, to the eruption being occasionally tardy in evincing itself; and, as regards the treatment, his experience leads him to attach great importance to the early exhibition of stimulants in these cases.—*Dublin Hospital Gazette.*

